# Controversial Effects of D-Amino Acid Oxidase Activator (DAOA)/G72 on D-Amino Acid Oxidase (DAO) Activity in Human Neuronal, Astrocyte and Kidney Cell Lines: The N-methyl D-aspartate (NMDA) Receptor Hypofunction Point of View

**DOI:** 10.3389/fnmol.2017.00342

**Published:** 2017-10-24

**Authors:** Vinita Jagannath, Zacharias Faidon Brotzakis, Michele Parrinello, Susanne Walitza, Edna Grünblatt

**Affiliations:** ^1^Molecular and Neurobiochemistry Laboratory, Centre for Child and Adolescent Psychiatry Research, Department of Child and Adolescent Psychiatry and Psychotherapy, University Hospital of Psychiatry Zurich, University of Zurich, Zurich, Switzerland; ^2^Department of Chemistry and Applied Biosciences, Eidgenössische Technische Hochschule (ETH) Zurich, Zurich, Switzerland; ^3^Faculty of Informatics, Institute of Computational Science, Università della Svizzera Italiana (USI), Lugano, Switzerland; ^4^Neuroscience Center Zurich, University of Zurich and ETH Zurich, Zurich, Switzerland; ^5^Zurich Center for Integrative Human Physiology, University of Zurich, Zurich, Switzerland

**Keywords:** DAO/DAAO, DAOA/G72, NMDA receptor, SH-SY5Y, 1321N1, HEK293

## Abstract

Dysfunction of D-amino acid oxidase (*DAO*) and DAO activator (*DAOA*)/*G72* genes have been linked to neuropsychiatric disorders. The glutamate hypothesis of schizophrenia has proposed that increased DAO activity leads to decreased D-serine, which subsequently may lead to N-methyl-D-aspartate (NMDA) receptor hypofunction. It has been shown that DAOA binds to DAO and increases its activity. However, there are also studies showing DAOA decreases DAO activity. Thus, the effect of DAOA on DAO is controversial. We aimed to understand the effect of DAOA on DAO activity in neuron-like (SH-SY5Y), astrocyte-like (1321N1) and kidney-like (HEK293) human cell lines. DAO activity was measured based on the release of hydrogen peroxide and its interaction with Amplex Red reagent. We found that DAOA increases DAO activity only in HEK293 cells, but has no effect on DAO activity in SH-SY5Y and 1321N1 cells. This might be because of different signaling pathways, or due to lower DAO and DAOA expression in SH-SY5Y and 1321N1 cells compared to HEK293 cells, but also due to different compartmentalization of the proteins. The lower DAO and DAOA expression in neuron-like SH-SY5Y and astrocyte-like 1321N1 cells might be due to tightly regulated expression, as previously reported in the human post-mortem brain. Our simulation experiments to demonstrate the interaction between DAOA and human DAO (hDAO) showed that hDAO holoenzyme [hDAO with flavine adenine dinucleotide (FAD)] becomes more flexible and misfolded in the presence of DAOA, whereas DAOA had no effect on hDAO apoprotein (hDAO without FAD), which indicate that DAOA inactivates hDAO holoenzyme. Furthermore, patch-clamp analysis demonstrated no effect of DAOA on NMDA receptor activity in NR1/NR2A HEK293 cells. In summary, the interaction between DAO and DAOA seems to be cell type and its biochemical characteristics dependent which still needs to be elucidated.

## Introduction

The human D-amino acid oxidase (*DAO*/*DAAO*) gene is located at chromosome 12q24, and encodes for a ~39 kDa protein of 347 amino acids (Verrall et al., 2010). The human DAO activator (*DAOA*)/*G72* gene is a primate specific gene located at chromosome 13q33, and encodes for a ~20 kDa protein of 153 amino acids (Benzel et al., 2008). Previous studies have shown evidence for significant association of nucleotide variations at *DAO* and *DAOA* locus with schizophrenia and bipolar disorder (Detera-Wadleigh and McMahon, 2006; Allen et al., 2008; Prata et al., 2008; Gatt et al., 2015). Although the effects of these *DAO* and *DAOA* nucleotide variations on their mRNA and protein expression in schizophrenia is not yet studied, these genes still remain as candidate genes for schizophrenia because of their role in the glutamatergic signaling.

DAO is a peroxisomal flavoenzyme. It catalyzes the oxidation of D-amino acids through concomitant reduction of flavine adenine dinucleotide (FAD), producing corresponding imino acid, which is then hydrolyzed to yield ammonia and corresponding α-keto acid. During FAD reoxidation, hydrogen peroxide is produced (Verrall et al., 2010). FAD binding is weaker in human DAO (hDAO) compared to DAO from other species, which provides hDAO a potential means to regulate DAO activity (Caldinelli et al., 2009). DAO protein and enzymatic activity is present mainly in the human kidney, liver and brain (Sasabe et al., 2014; Uhlén et al., 2015; Jagannath et al., 2017). In the human brain, its main substrate is D-serine (Pollegioni et al., 2007; Sacchi et al., 2012). D-serine serves as a co-agonist at the glycine site of the N-methyl-D-aspartate (NMDA) receptor. NMDA receptors are glutamate ionotropic receptors which require both glutamate and co-agonist (D-serine or glycine) to function normally (Panatier et al., 2006; Henneberger et al., 2010; Papouin et al., 2012). Thus, DAO can regulate the function of NMDA receptors via D-serine breakdown.

The glutamate hypothesis of schizophrenia is based on the NMDA receptor hypofunction (Stahl, 2007). One possible explanation for NMDA receptor hypofunction theory proposed in schizophrenia is increased DAO activity leading to decreased D-serine which subsequently causes hypofunction of the NMDA receptors. Chumakov et al. (2002) showed that DAOA binds to DAO and increases its activity. However, Sacchi et al. (2008) showed that DAOA binds to DAO and decreases its activity. Furthermore, Kvajo et al. (2008) showed that there was no interaction between DAO and DAOA. Thus, the effect of DAOA on DAO is controversial, and yet to be elucidated. Previous *in vitro* studies have shown that DAOA localizes in mitochondria and causes mitochondrial dysfunction (Kvajo et al., 2008; Sacchi et al., 2011; Otte et al., 2014). Thus, the exact function of DAOA is not yet completely understood. Since the microscopic interactions between DAO and DAOA may play an additional role in DAO activation, *in silico* Molecular Dynamics (MD) simulations may contribute in understanding the role of DAOA on DAO activity. Thus, this approach may contribute to the *in vitro* insight into the nature and interactions between these two proteins. In particular, we performed simulations of different DAO (apoprotein, holoenzyme) forms with and without DAOA, and studied the stability of DAO in terms of its flexibility, thus verifying one of the above DAO and DAOA interaction hypotheses.

DAO and DAOA proteins are detected in the human brain with brain region specificity which is tightly regulated (Jagannath et al., 2017). DAO has been shown to interact with DAOA in glial cells (Sacchi et al., 2016). However, DAO is not solely glial, but it has been reported to be also expressed in neurons (Verrall et al., 2010). In our study, we used neuron-like SH-SY5Y, astrocyte-like 1321N1 and kidney-like HEK293 cells to understand the interaction between DAO and DAOA proteins. We overexpressed DAO and DAOA in these human cell lines because of the lower endogenous expression of *DAO* and *DAOA* mRNA in these cells (Uhlén et al., 2015; Rouillard et al., 2016).

Since the interaction between DAO and DAOA is still not clear, we aimed: (1) to understand the effect of DAOA on DAO activity in different types of human cell lines; and (2) to understand the effect of DAOA on NMDA receptor activity in human cell lines.

## Materials and Methods

### Cell Culture

The human embryonic kidney HEK293 cell line (85120602, Sigma-Aldrich) and human astrocytoma 1321N1 cell line (86030402, Sigma-Aldrich) were cultured in Dulbecco’s Modified Eagle Medium (DMEM; 41966029, ThermoFisher scientific) containing 10% fetal bovine serum (FBS; 10270106, ThermoFisher scientific), and incubated at 37°C in a humidified atmosphere of 5% CO_2_. The human neuroblastoma SH-SY5Y cell line (CRL-2266, ATCC) was cultured in a 1:1 mixture of DMEM and DMEM/F12 (D8437, Sigma-Aldrich) supplemented with 10% FBS, and incubated at 37°C in a humidified atmosphere of 5% CO_2_.

### Transient Transfection

The human LG72 cDNA (GenBank sequence: AY138546) cloned into the pEGFPN1 vector was a generous gift from the Institute of Molecular Psychiatry, University of Bonn, Germany (Otte et al., 2011), and the pEGFPN1 construct (Takara Clontech) was used as a control plasmid. The human DAO cDNA (GenBank sequence: BC029057) cloned into pCMV3-c-Myc vector (HG13372-CM, Sino Biological) and the pCMV3-c-Myc vector (CV014, Sino Biological) were used for transfection. For all experiments except cell viability, HEK293, 1321N1 and SH-SY5Y cells were seeded into 6-well plates (3335, Corning) at a density of 2 × 10^5^ cells, 5 × 10^5^ and 1 × 10^6^ cells in 1 mL growth medium, respectively. The cells were allowed to adhere for 24 h. The cells were transfected with 5 μg of above-mentioned plasmids using Xfect transfection reagent (631317, Takara Clontech) according to manufacturer’s guidelines. The growth medium was exchanged 4 h after transfection. For all experiments, cells were incubated for 48 h following transfection.

### DAO Activity Assay

In order to evaluate whether DAOA affects DAO activity, DAO activity was determined based on the estimation of hydrogen peroxide formation as previously described (Sikka et al., 2010). The transfected cells were harvested with 0.05% Trypsin-EDTA (25300054, ThermoFisher Scientific) solution, transferred to a 15 mL falcon tube and centrifuged for 5 min at 12,000 × *g*. The cell pellets were homogenized in 100 μL of cold sodium phosphate buffer (50 mM Na_2_HPO_4_, pH 7.4) with 5 mm stainless steel beads (69989, Qiagen) using the TissueLyser II (Qiagen). In a 384-well optical-bottom plate (142761, ThermoFisher Scientific), 10 μL of homogenate was added to a solution of 100 μM Amplex red (A36006, ThermoFisher Scientific), 0.25 U/mL horseradish peroxidase (10108090001, Sigma-Aldrich), 50 mM D-serine (S4250, Sigma-Aldrich) and with or without 10 μM FAD (F6625, Sigma-Aldrich) in a total volume of 20 μl of sodium phosphate buffer. After 1 h of incubation at 37°C, the fluorescence was measured with a Mithras^2^ LB 943 Multimode Reader (Berthold technologies) using 544 nm excitation (slit 22 nm) and 590 nm emission (slit 20 nm) filters. On each plate, we included 3 controls namely, a control without cell homogenate, a control without D-serine and a control inhibiting DAO activity with a DAO inhibitor, 100 μM 6-methyl-benzo[d]isoxazol-3-ol (027-640-512, MolPort). The fluorescence values of cell homogenates were calculated by subtracting their fluorescence values from fluorescence values of control without D-serine. As the wells transfected with control plasmids (pCMV3-c-Myc and pEGFPN1) showed fluorescence similar to the control without D-serine, fluorescence values of cells transfected with LG72 and DAO plasmids were used for analysis.

### Cell Viability Assay

We evaluated cell viability to make sure that the results obtained for DAO activity were not biased by the sensitivity to different cell lines to transfection. The viability of transfected cells was determined using CellTiter 96^®^ AQueous One Solution Cell Proliferation Assay (G3582, Promega) according to manufacturer’s guidelines. HEK293, 1321N1 and SH-SY5Y cells were seeded into 96-well plates (83.3925, Sarstedt) at a density of 2 × 10^4^ cells, 5 × 10^4^ and 1 × 10^5^ cells in 100 μL growth medium, respectively. They were allowed to adhere for 24 h and were transfected with 1.2 μg of plasmids using Xfect transfection reagent (631317, Takara Clontech) according to manufacturer’s guidelines. The growth medium was exchanged 4 h after transfection. Each transfection condition was analyzed in triplicates. The cells were incubated for 48 h following transfection. Then, 20 μl of CellTiter 96^®^ AQueous One Solution Reagent was added to each well and incubated at 37°C for 4 h. The absorbance was measured at 490 nm using Mithras^2^ LB 943 Multimode Reader (Berthold Technologies). The absorbance values were corrected by subtracting the average absorbance from the control wells with no cells.

### Simulation Setup for hDAO and DAOA Interactions

In this work, MD simulations were performed. All MD simulations were carried out using the GROMACS software (Abraham et al., 2015). In all simulations, the AMBER-14SB and the TIP3P forcefields (Jorgensen et al., 1983; Maier et al., 2015) were used for the protein and water, respectively. The simulated conditions were ambient, at a constant temperature of 300 K, pressure of 1 atm and concentration (NPT ensemble). The simulated systems were: hDAO without FAD in water, FAD/hDAO complex in water, FAD/hDAO/DAOA complex in water and hDAO/DAOA complex in water.

#### hDAO Without FAD (apoprotein)

The hDAO monomer structure was extracted from the dimer protein data bank (PDB) structure of hDAO (PDB: 2E48), and the FAD ligand was removed.

#### FAD/hDAO (holoenzyme) Complex

The structure of this complex was obtained by extracting the hDAO monomer structure from the dimer PDB structure of hDAO while retaining the FAD ligand. The topology of the complex was obtained using the AMBER tools and the AMBER-14SB forcefield, after having created the FAD topology. The FAD topology was obtained using the gaff forcefield, followed by a geometry optimization using Gaussian package and the B3LYP/6-31G, constructing the electrostatic potential surface and assigning charges to atoms using RESP (Bayly et al., 1993).

#### FAD/hDAO/DAOA Complex

The structure of this complex was obtained by using the patchdock docking server (Schneidman-Duhovny et al., 2005), and adding constraints on the dimer interface so that DAOA residues 138, 139, 140, 141, 142, 143, 144, 145, 146, 147, 148, 149, 150, 151, 152, 153 were within 0.4 nm of any amino acids within 1 nm of hDAO amino acids 37, 138, 163, 185, 188, 191, 193, 194, 195, 196. These contacts have been found to occur in the hDAO-DAOA complex (Chang et al., 2014). As mentioned earlier, building the FAD topology and using AMBER tools to combine the topology of FAD, hDAO and G72 protein, the final complexes topology was obtained. Simulations were performed using the structure of the best binding pose.

#### hDAO/DAOA Complex

The procedure for constructing the structure of this complex was the same as the above ones, except the topology of the complex was obtained by using the AMBER tools for complex’s structure.

#### DAOA Protein

Since there was no available crystal structure for the human DAOA protein, we obtained its structure by using homology modeling of the protein sequence with accession number AAN08432. The homology modeling was performed using the I-TAISER server (Yang and Zhang, 2015). The best predicted homolog structure was used as the DAOA protein.

### NMDA Receptor Currents Using Whole-Cell Patch Clamp Recording

HEK293 cells stably expressing NMDA receptor subunits NR1 and NR2A (NR1/NR2A HEK293 cells; B’SYS, Switzerland) were used. They were cultured in DMEM/F12 (D8437, Sigma-Aldrich) supplemented with 10% FBS (RNBF7902, ThermoFisher scientific), 1% penicillin/streptomycin (10378016, ThermoFisher scientific), 100 μg/mL hygromycin (10687010, ThermoFisher scientific), 15 μg/mL blasticidin (A1113903, ThermoFisher scientific), 1 μg/mL puromycin (A1113803, ThermoFisher scientific) and incubated at 37°C in a humidified atmosphere of 5% CO_2_. The cells (2 × 10^5^ cells/well) were seeded onto Poly-L-Lysine (P4832, Sigma-Aldrich) coated coverslips placed in 6-well plates (92412, TPP) and were allowed to adhere for 24 h. The cells were transfected with 5 μg of LG72 and control pEGFPN1 plasmids using Xfect transfection reagent according to manufacturer’s guidelines. The growth medium was exchanged 4 h after transfection. Expression of NR1 and NR2A subunits in the cells were induced 24 h after transfection using 2.5 μg/mL tetracycline (T7660, Sigma-Aldrich) in the presence of 50 μM AP5 (ab120003, abcam). The whole-cell patch clamp recording was performed 48 h after transfection at room temperature using Amplifier EPC-10 and Preamplifier EPC-10 (both, HEKA Electronics). For recording, the coverslips with transfected or non-transfected cells were placed in a 35-mm culture dish containing 2 mL of standard bath solution (137 mM sodium chloride, 4 mM potassium chloride, 1.8 mM calcium chloride, 1 mM magnesium chloride, 10 mM HEPES, 10 mM D-Glucose, pH 7.4 adjusted with sodium hydroxide). The transfected cells were identified visually by green florescent protein (GFP) fluorescence signal using inverted microscope IM (Zeiss). The patch pipettes were filled with pipette solution (130 mM potassium chloride, 1 mM magnesium chloride, 5 mM magnesium-ATP, 10 mM HEPES, 5 mM EGTA, pH 7.2 adjusted with potassium hydroxide). After formation of Gigaohm seal between the patch pipettes and single cell (pipette resistance range: 2.5 MΩ to 6.0 MΩ; seal resistance range: >1 GΩ), the cell membrane across the pipette tip was ruptured to ensure electrical access to the cell interior (whole-cell patch configuration). In case of poor seal quality, the process of seal formation was repeated with a different cell and a new pipette. As soon as a stable seal was established, magnesium-free bath solution (137 mM sodium chloride, 4 mM potassium chloride, 2.8 mM calcium chloride, 10 mM HEPES, 10 mM D-Glucose, 0.02% Cremophor, pH 7.4 adjusted with sodium hydroxide) was perfused and NMDA inward currents were measured upon application of submaximal concentrations of NMDA (100 μM; M3262, Sigma-Aldrich)/Glycine (5 μM; 410225, Sigma-Aldrich) to patch-clamped cells for 4 s. If the current was too unstable for measurement, another cell was recorded. During the entire experiment, the membrane potential of the cell was clamped at −80 mV. We used only data from cells treated with the complete application protocol for analysis. At least five cells were recorded per condition and three independent experiments were performed. Data was acquired using PatchMaster (HEKA Electronics, version v2x73_2) software and analyzed using Microsoft Excel 2003 software. The NMDA peak current density was calculated by dividing means of peak current amplitudes (pA) from at least 5 cells by cell capacitance (pF). The NMDA mean current density was calculated by dividing means of steady state current amplitudes (pA) from at least five cells by cell capacitance (pF).

### Statistical Analysis

The results presented in this study are from at least three independent experiments. IBM^®^ SPSS^®^ Statistics (version 23) software was used for statistical analysis. Shapiro-Wilk test with Lilliefors significance correction was used to assess the normality of the distribution of NMDA receptor current, DAO activity and cell viability data. As the NMDA receptor current data was normally distributed, we used one-way analysis of variance (ANOVA) followed by *post hoc* Bonferroni test to analyze the difference between the groups, and *p* < 0.05 was considered statistically significant. As the DAO activity and cell viability data was also normally distributed, we used independent-samples *t*-test to analyze the difference between two groups, and *p* < 0.05 was considered statistically significant. GraphPad Prism software (version 6.01) was used to plot the graphs.

## Results

### DAO Activity and Cell Viability in SH-SY5Y Cells

**Figure 1:**
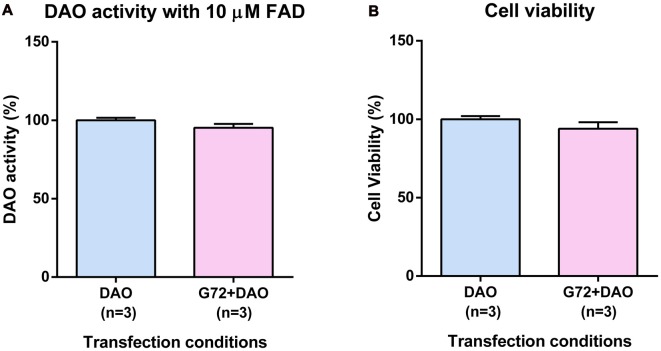
D-amino acid oxidase (DAO) activity and cell viability in SH-SY5Y cells. **(A)** DAO activity with the addition of 10 μM flavine adenine dinucleotide (FAD) across two transfection conditions: DAO and G72+DAO. **(B)** Cell viability across two transfection conditions: DAO and G72+DAO. Data is presented as bar graphs with mean ± SEM. Differences between the two transfection conditions was assessed by the independent-samples *t*-test.

To understand the effect of DAOA on DAO activity, SH-SY5Y cells were transfected with either DAO plasmid alone or both DAO and G72 plasmids. To achieve DAO-activity signal in SH-SY5Y cells, the addition of 10 μM FAD was necessary. There were no significant differences in DAO activity between the two transfection conditions, namely DAO and G72+DAO (Figure 1A). In order to verify that the non-significant differences in DAO activity between single (DAO) and double transfection (G72+DAO) conditions is not due to cell death, we performed cell viability assay. There were no significant differences in cell viability between the two transfection conditions (Figure 1B).

### DAO Activity and Cell Viability in 1321N1 Cells

**Figure 2:**
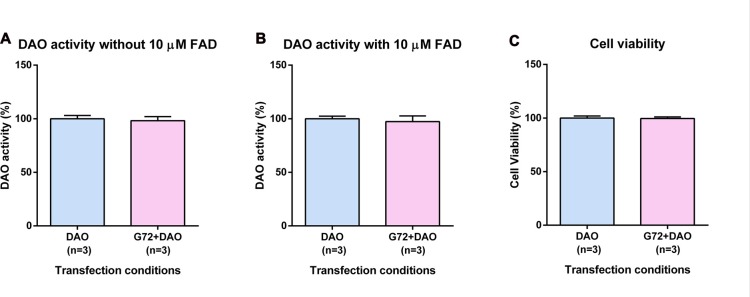
DAO activity and cell viability in 1321N1 cells. **(A)** DAO activity without the addition of 10 μM FAD across two transfection conditions: DAO and G72+DAO. **(B)** DAO activity with the addition of 10 μM FAD across two transfection conditions: DAO and G72+DAO. **(C)** Cell viability across two transfection conditions: DAO and G72+DAO. Data is presented as bar graphs with mean ± SEM. Differences between the two transfection conditions was assessed by the independent-samples *t*-test.

To verify the results obtained in SH-SY5Y neuroblastoma cells with neuron-like phenotype, we retested effects of DAOA on DAO activity in the human 1321N1 cells which have an astrocyte-like phenotype. Again, we single and double transfected 1321N1 cells with DAO plasmid and both DAO and G72 plasmids, respectively. In contrast to SH-SY5Y cells, DAO activity assay in 1321N1 cells worked both with and without addition of 10 μM FAD. We did not find any significant differences in DAO activity without (Figure 2A) and with (Figure 2B) addition of 10 μM FAD between the two transfection conditions as well as no significant differences in cell viability between the two transfection conditions (Figure 2C).

### DAO Activity and Cell Viability in HEK293 Cells

**Figure 3:**
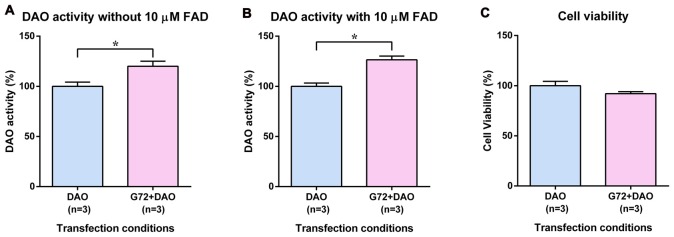
DAO activity and cell viability in HEK293 cells. **(A)** DAO activity without the addition of 10 μM FAD across two transfection conditions: DAO and G72+DAO. **(B)** DAO activity with the addition of 10 μM FAD across two transfection conditions: DAO and G72+DAO. **(C)** Cell viability across two transfection conditions: DAO and G72+DAO. Data is presented as bar graphs with mean ± SEM. Differences between the two transfection conditions was assessed by the independent-samples *t*-test (*p < 0.05).

To test the tissue related specificity of the results obtained in neuron-glia like cell lines, we determined the effect of DAOA on DAO activity also in human HEK293 cells with kidney epithelium-like phenotype. HEK293 cells were single and double transfected with DAO plasmid and with both DAO and G72 plasmids, respectively to understand the effect of DAOA on DAO activity. Similar to 1321N1 cells, DAO activity assay in HEK293 cells worked with and without addition of 10 μM FAD. In contrast to SH-SY5Y and 1321N1 cells, DAO activity without (*t* = 3.024, *df* = 10, *p* = 0.013; Figure 3A) and with (*t* = 5.305, *df* = 10, *p* = 0.0003; Figure 3B) addition of 10 μM FAD was significantly increased in double transfected HEK293 cells (G72+DAO) compared to the single transfected (DAO) HEK293 cells. The cell viability assay was performed to confirm that the differences in DAO activity are not due to cell death. There were no significant differences in cell viability between the two transfection conditions (Figure 3C).

Although DAO protein was detected at the expected size of 40 kDa in pEGFPN1+pCMV3-c-Myc and pCMV3-c-Myc transfection conditions in all three cell lines (data not shown), we could not detect DAO activity signal in these transfection conditions which might be because the endogenous DAO enzyme is in FAD unbound inactive state.

### *In Silico* Interactions between hDAO and DAOA

**Figure 4:**
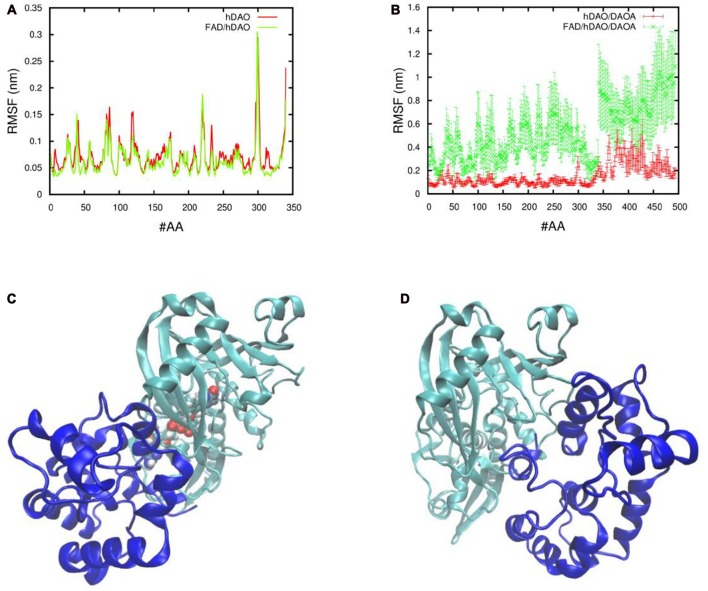
Fluctuations and 3D conformations of human DAO (hDAO) holoenzyme and apoprotein complex with DAO activator (DAOA). Room mean square fluctuations of the Cα atom per amino acid (#AA) of **(A)** the hDAO without FAD (red), and the FAD/hDAO complex (green), and **(B)** the hDAO/DAOA complex (red) and FAD/hDAO/DAOA complex (green). Note that the amino acid index 0-340 corresponds to the hDAO Cα atoms, whereas the index 341-494 corresponds to the Cα atoms of DAOA amino acids. 3D conformations taken from Molecular Dynamics (MD) simulation of holoenzyme FAD/hDAO/DAOA complex **(C)** and apoprotein hDAO/DAOA complex **(D)** with hDAO in green color and DAOA in blue color.

In order to understand the effect of DAOA protein on the holoenzyme (FAD/hDAO) and apoprotein (hDAO without FAD) form of the hDAO, the fluctuations of the Cα atoms of each amino acid were determined. We found that there was a slight decrease in FAD/hDAO complex flexibility compared to hDAO without FAD (Figure 4A). Our simulations show that upon FAD/hDAO/DAOA complex formation, FAD/hDAO become much more flexible (Figure 4B) and misfolded (Figure 4C) than FAD/hDAO complex without DAOA. Contrarily, the apoprotein form of the hDAO when in complex with DAOA, shows much less flexibility (Figure 4B), and retains its folded structure (Figure 4D).

### NMDA Receptor Currents in NR1/NR2A HEK293 Cells

To understand the effect of DAOA on NMDA receptor activity, NR1/NR2A HEK293 cells were transfected with G72 plasmid. There were no significant differences in mean NMDA receptor current (Supplementary Figure S1A) or in peak NMDA receptor current (Supplementary Figure S1B) between all three conditions namely, G72 transfected, pEGFPN1 plasmid transfected and non-transfected.

### *DAO* and *DAOA* mRNA Expression in Transfected SH-SY5Y, 1321N1 and HEK293 Cells

**Figure 5:**
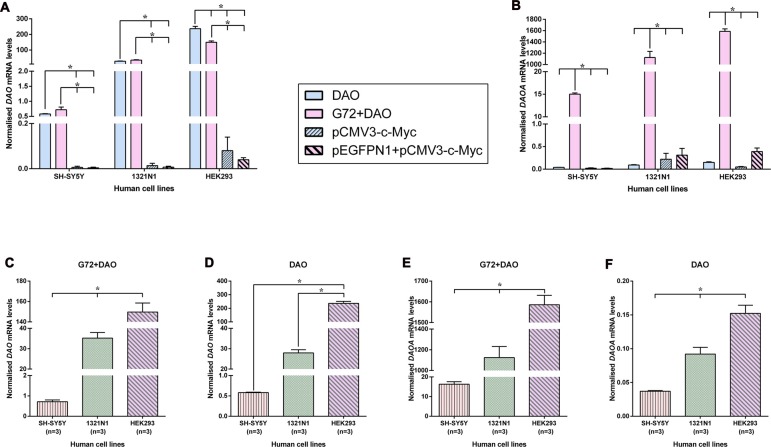
*DAO* and *DAOA* mRNA levels in SH-SY5Y, 1321N1 and HEK293 cells across different transfection conditions. *DAO* **(A)** and *DAOA* **(B)** mRNA levels across four transfection conditions: DAO, G72+DAO, pCMV3-c-Myc and pEGFPN1+ pCMV3-c-Myc in all three cell lines. *DAO* mRNA levels in two transfection conditions: G72+DAO **(C)** and DAO **(D)**. *DAOA* mRNA levels in two transfection conditions: G72+DAO **(E)** and DAO **(F)**. Data is presented as bar graphs with mean ± SEM. Differences in *DAO* and *DAOA* mRNA levels between four transfection conditions, and also between the three cell lines was assessed by one-way analysis of variance (ANOVA) followed by *post hoc* Bonferroni test (*p < 0.05).

In order to verify and compare the expression of endogenous and overexpressed *DAO* and *DAOA* mRNA in transfected SH-SY5Y, 1321N1 and HEK293 cells, we measured the *DAO* and *DAOA* mRNA levels using qRT-PCR. We found that there were no significant differences in *DAO* mRNA levels between the double transfection (G72+DAO) and single transfection (DAO) conditions in SH-SY5Y and 1321N1 cells, but a significantly lower endogenous *DAO* mRNA levels in pEGFPN1+pCMV3-c-Myc and pCMV3-c-Myc transfected compared to the G72+DAO and DAO transfected SH-SY5Y (*F* = 74.91, *df* = 3, *p* < 0.0001) and 1321N1 cells (*F* = 135.98, *df* = 3, *p* < 0.0001; Figure 5A). There was a significant decrease in *DAO* mRNA levels in double transfected (G72+DAO) compared to the single transfected (DAO) HEK293 cells (Bonferroni test *p* = 0.001), and endogenous *DAO* mRNA levels were significantly lower in pEGFPN1+pCMV3-c-Myc and pCMV3-c-Myc transfected HEK293 cells compared to G72+DAO and DAO transfected HEK293 cells (*F* = 182.58, *df* = 3, *p* < 0.0001; Figure 5A). We found that the endogenous *DAOA* mRNA levels were significantly lower in DAO, pEGFPN1+pCMV3-c-Myc, pCMV3-c-Myc transfected than G72+DAO transfected SH-SY5Y (*F* = 3656.23, *df* = 3, *p* < 0.0001), 1321N1 (*F* = 112.39, *df* = 3, *p* < 0.0001) and HEK293 cells (*F* = 858.86, *df* = 3, *p* < 0.0001; Figure 5B). There were significant differences in *DAO* mRNA levels between the three cell lines in G72+DAO transfection condition (*F* = 213.76, *df* = 2, *p* < 0.0001; Figure 5C), and *DAO* mRNA levels were the highest in HEK293 cells compared to the 1321N1 and SH-SY5Y cells in DAO transfection condition (*F* = 222.68, *df* = 2, *p* < 0.0001; Figure 5D). We found that there were significant differences in *DAOA* mRNA levels between the three cell lines both in G72+DAO (*F* = 164.83, *df* = 2, *p* < 0.0001; Figure 5E) and DAO (*F* = 56.35, *df* = 2, *p* < 0.0001; Figure 5F) transfection conditions. *DAOA* mRNA levels were the highest in HEK293 cells followed by intermediate levels in 1321N1 cells and low levels in SH-SY5Y cells.

### DAO and DAOA Protein Expression in Transfected SH-SY5Y, 1321N1, and HEK293 Cells

**Figure 6:**
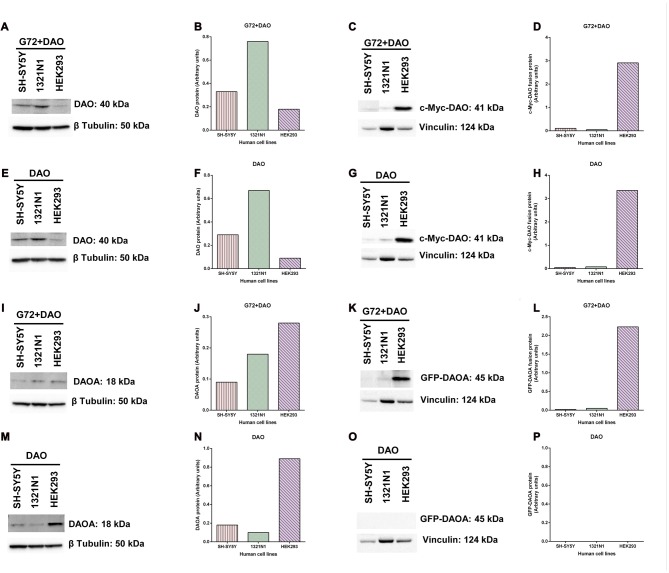
DAO and DAOA protein levels in SH-SY5Y, 1321N1 and HEK293 cells. Representative western blot images for DAO protein expression levels in three cell lines across two transfection conditions: G72+DAO **(A)** and DAO **(E)**, and DAO protein detected at an expected size of 40 kDa. DAO protein levels (arbitrary units) in three cells lines across two transfection conditions: G72+DAO **(B)** and DAO **(F)**. Representative western blot images for c-Myc-DAO fusion protein expression levels in three cell lines across two transfection conditions: G72+DAO **(C)** and DAO **(G)**, and c-Myc-DAO fusion protein detected at an expected size of 41 kDa. c-Myc-DAO protein levels (arbitrary units) in three cells lines across two transfection conditions: G72+DAO **(D)** and DAO **(H)**. Representative western blot images for DAOA protein expression levels in three cell lines across two transfection conditions: G72+DAO **(I)** and DAO **(M)**, and DAOA protein detected at an expected size of 18 kDa. DAOA protein levels (arbitrary units) in three cells lines across two transfection conditions: G72+DAO **(J)** and DAO **(N)**. Representative western blot images for green florescent protein (GFP)-DAOA fusion protein expression levels in three cell lines across two transfection conditions: G72+DAO **(K)** and DAO **(O)**, and GFP-DAOA fusion protein detected at an expected size of 45 kDa. GFP-DAOA protein levels (arbitrary units) in three cells lines across two transfection conditions: G72+DAO **(L)** and DAO **(P)**.

To verify and compare the overexpression of DAO and DAOA proteins in transfected SH-SY5Y, 1321N1 and HEK293 cells, we determined DAO and DAOA proteins using western blot. DAO protein was detected in all three cell lines at the expected size of 40 kDa in G72+DAO (Figure 6A) and DAO (Figure 6E) transfection conditions. DAO protein levels were the highest in 1321N1 cells compared to HEK293 and SH-SY5Y cells in G72+DAO (Figure 6B) and DAO (Figure 6F) transfection conditions. We detected c-Myc-DAO fusion protein in all three cell lines at the expected size of 41 kDa in G72+DAO (Figure 6C) and DAO (Figure 6G) transfection conditions. HEK293 cells had highest c-Myc-DAO fusion protein levels compared to 1321N1 and SH-SY5Y cells in G72+DAO (Figure 6D) and DAO (Figure 6H) transfection conditions. DAOA protein was detected in all three cell lines at the expected size of 18 kDa in G72+DAO (Figure 6I) and DAO (Figure 6M) transfection conditions. DAOA protein levels were the highest in HEK293 cells compared to 1321N1 and SH-SY5Y cells in G72+DAO (Figure 6J) and DAO (Figure 6N) transfection conditions. GFP-DAOA fusion protein was detected in all three cell lines at the expected size of 45 kDa in the double transfection with G72+DAO (Figure 6K), but not in the single transfection with DAO (Figures 6O,P). GFP-DAOA fusion protein levels were the highest in HEK293 cells compared to 1321N1 and SH-SY5Y cells in G72+DAO (Figure 6L) transfection condition.

## Discussion

*DAO* and *DAOA* genes are alleged to be involved in pathophysiology of neuropsychiatric disorders such as schizophrenia and bipolar disorder (Detera-Wadleigh and McMahon, 2006; Allen et al., 2008; Prata et al., 2008; Gatt et al., 2015), but the interactions between these genes remains unclear to date. There are several lines of evidence in humans suggesting NMDA receptor hypofunction in the pathophysiology of schizophrenia. Healthy controls manifested primary symptoms of schizophrenia on administration of NMDA receptor antagonists such as phencyclidine and ketamine (Anis et al., 1983; Krystal et al., 1994). Patients with NMDA receptor specific antibodies were reported to be associated with severe psychosis (Dalmau et al., 2008). A study revealed reduced expression of obligatory NMDA receptor subunit NR1 in post-mortem brains of schizophrenia patients (Weickert et al., 2013), and reduced D-serine in cerebrospinal fluid of schizophrenia patients (Hashimoto et al., 2005). Studies have also shown increased DAO activity in brains of schizophrenia patients compared to the healthy controls (Kapoor et al., 2006; Burnet et al., 2008; Madeira et al., 2008). There is only one published study using single-photon emission computed tomography (SPECT) with NMDA receptor tracer [^123^I]CNS-1261 which showed lower NMDA receptor binding in left hippocampus of schizophrenia patients compared to healthy controls (Pilowsky et al., 2006), however this finding awaits confirmation. Although, the aforementioned studies provide evidence for NMDA receptor hypofunction in schizophrenia, it is still not clear what NMDA receptor hypofunction means at the molecular level. At present there are no drugs on the market to treat the negative symptoms and cognitive deficits of schizophrenia, and clinical trials with DAO inhibitors or D-serine have not shown a conclusive or strong effect in schizophrenia (Smith et al., 2010). Thus, there is an urgent need for new drugs for the treatment of negative symptoms and cognitive deficits of schizophrenia, which might be achieved by understanding the interaction between DAO and DAOA, and their subsequent effect on NMDA receptors. In this study, we aimed to understand the interaction between DAO and DAOA by determining the effect of DAOA on DAO and NMDA receptor activity in different types of human cell lines.

We chose three human cell lines namely, neuron-like SH-SY5Y, astrocyte-like 1321N1 cells, and kidney-like HEK293 cells to verify and compare the effects of DAOA on DAO activity. We found that DAOA increases the activity of DAO only in HEK293 cells, but not in the SH-SY5Y and 1321N1 cells. One possible reason for this result might be because of different signaling pathways in kidney-like HEK293 cells vs. neuron-like SH-SY5Y and astrocyte-like 1321N1 cells. Moreover, our previous post-mortem study (Jagannath et al., 2017) showed that there is a tight transcriptional regulation in DAO and DAOA expression in the human brain during development and aging, which might also be the reason that we found no effect of DAOA on DAO activity in the neuron-like SH-SY5Y and astrocyte-like 1321N1 cells. Chumakov et al. (2002) showed that increasing concentrations of recombinant human DAOA protein causes porcine DAO activation. In the current study, we found that HEK293 cells overexpressed *DAOA* mRNA significantly more than in SH-SY5Y and 1321N1 cells, which might explain why DAO activity increased only in HEK293 cells when co-transfected with DAOA. A study conducted using recombinant hDAO and DAOA proteins isolated from *E.coli* found that DAOA increases DAO activity (Chang et al., 2014), but a recent study using recombinant hDAO and DAOA proteins isolated from *E.coli* found that DAOA decreases DAO activity (Birolo et al., 2016). Moreover, another study found that DAOA even decreases DAO activity in human glioblastoma U87 cell line (Sacchi et al., 2008). However, following the last result, a study conducted in human glioblastoma U251 cell line, reported that DAOA has no effect on DAO activity (Kvajo et al., 2008). All these studies performed the DAO activity assay based on the detection of hydrogen peroxide released from oxidation of D-amino acids by the DAO enzyme. The differences in results found in these studies might be because of substrate, cell lines and enzymatic reaction temperature used (see summary in Table 1). Chumakov et al. (2002) used recombinant human DAOA but recombinant porcine DAO to perform DAO activity assay with D-serine as substrate and the enzymatic reaction was performed at 30°C. However, two independent research groups used D-alanine as substrate and the assay was performed at 25°C (Chang et al., 2014; Birolo et al., 2016). Furthermore, another two groups conducted the DAO activity assay at room temperature, but Kvajo et al. (2008) used D-proline as substrate, while Sacchi et al. (2008) used D-serine as substrate. In our study, we used D-serine as substrate and performed the assay at 37°C, which corresponds to the human body temperature. The DAO activity assay conducted with D-proline as substrate (Kvajo et al., 2008) can be biased because D-proline is also a substrate of human D-aspartate oxidase (Katane et al., 2015). A study found that DAO shows lower catalytic efficiency and substrate affinity on the physiological substrate D-serine compared to D-alanine (Molla et al., 2006b), which might explain the discrepancies found between studies using these substrates. We found that DAO activity assay worked in 1321N1 and HEK293 cells even without the addition of 10 μM FAD, which might be because of endogenous production of FAD by these cells. Indeed, FAD co-factor binding was reported to be essential for the DAO enzyme activity (Molla et al., 2006b). Nevertheless, we found that addition of 10 μM FAD had no effect on the results obtained comparing to those obtained without the addition of 10 μM FAD in these two cell lines. In studies using recombinant DAO and DAOA proteins, two of the studies, which did not use FAD in DAO activity assay, found DAOA to be a DAO activator (Chumakov et al., 2002; Chang et al., 2014). However, a recent study with human recombinant DAO and DAOA proteins, where they added FAD in DAO activity assay found DAOA to be a DAO inhibitor (Birolo et al., 2016). Thus, additional studies in a more realistic conditions i.e., human induced pluripotent stem cells (hiPSC) derived neurons and glia using gene editing tools will be required to conclusively determine whether DAOA is a DAO activator or inhibitor.

**Table 1:** Summary of publications to date showing effect of D-amino acid oxidase activator (DAOA) on DAO activity.

Serial No.	Publications	Species of DAO protein	Species of DAOA protein	Recombinant proteins or types of cell lines used	Substrate used	DAO activity assay method used	FAD added (+) or not (−)	Enzymatic reaction incubation temperature	Effect of DAOA on DAO activity
1	Chumakov et al. (2002)	Porcine	Human	*DAOA* cDNA was expressed in *E. coli* BL21(DE3) cells using the pET11b expression vector, and recombinant DAOA protein was isolated using chromatography, and DAO protein was purified from Sigma crude preparation	D-serine	o-dianisidine	+	30°C	Increases DAO activity
2	Chang et al. (2014)	Human	Human	*DAO* and *DAOA* cDNA were expressed in *E. coli* BL21(DE3)pLysS cells using the pET23a expression vector, and recombinant DAO and DAOA proteins were isolated using HisTrap FF column and column-refolding procedure (Molla et al., 2006a), respectively	D-alanine	o-phenylenediamine	−	25°C	Increases DAO activity
3	Birolo et al. (2016)	Human	Human	*DAO* and *DAOA* cDNA were expressed in *E. coli* BL21(DE3) cells using the pET11b expression vector, and recombinant DAO and DAOA proteins were isolated using anionic exchange chromatography on Sepharose FF column	D-alanine	Oxygen electrode	+	25°C	Decreases DAO activity
4	Sacchi et al. (2008)	Human	Human	Human glioblastoma U87 cell line	D-serine	Amplex Red	+	Room temperature	Decreases DAO activity
5	Kvajo et al. (2008)	Human	Human	Human glioblastoma U251 cell line	D-proline	Alexa-coupled tyramide	−	Room temperature	No effect on DAO activity
6	Current study	Human	Human	Human neuroblastoma SH-SY5Y cell line	D-serine	Amplex Red	+	37°C	No effect on DAO activity
				Human astrocytoma 1321N1 cell line	D-serine	Amplex Red	±	37°C	No effect on DAO activity
				Human embryonic kidney HEK293 cell line	D-serine	Amplex Red	±	37°C	Increases DAO activity

In our simulation experiments, we found that there is a slight decrease in FAD/hDAO (holoenzyme) complex flexibility compared to the hDAO without FAD (apoprotein) which indicates that the hDAO holoenzyme is more stable than the hDAO apoprotein. This result coincides with the previous spectroscopic evidence of hDAO holoenzyme having slightly higher melting temperature than the hDAO apoprotein (Caldinelli et al., 2009; Sacchi et al., 2012). Previous studies based on spectroscopy and proteolysis experiments have shown that DAOA changes the tertiary structure of FAD/hDAO and leads to decrease in hDAO holoenzyme form (Caldinelli et al., 2010), which is in line with our simulations that showed DAOA makes FAD/hDAO more flexible (i.e., less stable) and misfolded. However, our simulations showed that DAOA had no effect on hDAO apoprotein structure which corroborates with previous proteolysis experiments that showed hDAO apoprotein structure is not affected by DAOA (Caldinelli et al., 2010). The difference in flexibility of hDAO apoprotein and holoenzyme in complex with DAOA can be explained by the different binding positions of DAOA. DAOA binds to hDAO holoenzyme much closer to the FAD binding pocket, which causes an increase in the complex’s fluctuations (i.e., less stable), compared to the hDAO apoprotein where DAOA binds slightly farther away from the FAD binding pocket. Thus, our simulations indicate that DAOA inactivates hDAO holoenzyme and not the hDAO apoprotein. However, we did not observe the similar inactivating effect of DAOA on DAO activity in our cell lines which might be due to the fact that in our simulations we used only hDAO and DAOA proteins in an artificial system. Moreover, as indicated in our previous postmortem study (Jagannath et al., 2017), the tight regulation of DAO and DAOA in the human brain compared to the periphery might be an additional reason for the *in vitro* differences found in neuron-like and astrocyte-like cells compared to the kidney-like cells.

Accordingly, going one-step forward, we tested whether DAOA overexpression modulates NMDA receptor activity using induced NR1/NR2A HEK293 cells. In this study, we did not find an effect of DAOA on NMDA receptor currents in NR1/NR2A HEK293 cell line, despite our results showing DAOA increases DAO activity in HEK293 cells. This might be because of several reasons namely; the endogenous DAO (binding partner of DAOA) in NR1/NR2A HEK293 cell line is not in an active form leading to no effect on D-serine, unavailability of D-serine transporters in these NR1/NR2A HEK293 cells to transport the D-serine extracellularly for its action on NMDA receptors, and the DAOA transfected NR1/NR2A HEK293 cell line is an artificial model system which doesn’t recapitulate the human tripartite synapse and the complex signaling pathways of human neurons and glia. A study conducted in primary cultures from rat hippocampus using whole cell patch clamp technique showed that DAO inhibitors lead to increase in NMDA receptor mediated currents (Strick et al., 2011). To our knowledge, there are no articles showing the effect of DAOA on NMDA receptor currents *in vitro*. Thus, future experiments must be conducted, probably also in a more complex cellular system, to understand the effect of DAOA on NMDA receptors.

In summary, we found that DAOA increases DAO activity only in kidney-like HEK293, but not in neuron-like SH-SY5Y and astrocyte-like 1321N1 cells. We also found no effect of DAOA on NMDA receptor currents in NR1/NR2A HEK293 cells. As our study was performed using human cell lines, in order to confirm the tight regulation of DAO and DAOA assumed in the human central nervous system (CNS), modeling the interactions between DAO and DAOA proteins in schizophrenia patient specific neuroglial culture i.e., hiPSC derived neurons and glia co-cultures and comparing them to healthy control derived neuroglial cultures might further help in settling the debate of DAOA effect on DAO and NMDA receptor activity.

## Author Contributions

VJ and EG designed the experiments. VJ performed *in vitro* experiments and analyzed data. ZFB performed *in silico* experiments and analyzed data. VJ drafted and revised the manuscript. MP, SW and EG reviewed the manuscript. All authors have approved the final manuscript.

## Conflict of Interest Statement

SW has received lecture honoraria from Eli-Lilly, Astra Zeneca, Shire, Opopharma in the last 5 years. Outside professional activities and interests are declared under the link of the University of Zurich www.uzh.ch/prof/ssl-dir/interessenbindungen/client/web. The other authors declare that the research was conducted in the absence of any commercial or financial relationships that could be construed as a potential conflict of interest.
