# Supplementary material for: Controversial Effects of D-Amino Acid Oxidase Activator (DAOA)/G72 on D-Amino Acid Oxidase (DAO) Activity in Human Neuronal, Astrocyte and Kidney Cell Lines: The N-methyl D-aspartate (NMDA) Receptor Hypofunction Point of View
Source: Front Mol Neurosci. 2017 Oct 24;10:342. doi: 10.3389/fnmol.2017.00342 (PMC5660716; doi:10.3389/fnmol.2017.00342)
Supplement: Supplementary file 1 [file Presentation_1.pdf]

frontiers

Supplementary Material

Controversial effects of D-amino acid oxidase activator (DAOA)/G72 on D-amino acid oxidase (DAO) activity in human neuronal, astrocyte, and kidney cell lines: The N-methyl D-aspartate (NMDA) receptor hypofunction point of view

Vinita Jagannath[1], Zacharias Faidon Brotzakis[4,5], Michele Parrinello[4,5], Susanne Walitza[1,2,3], Edna Grünblatt[1,2,3*]

*** Correspondence:** Edna Grünblatt: edna.gruenblatt@kjpd.uzh.ch

1 Supplementary Methods, Results, and Figures

1.1 Supplementary Methods

S1 RNA isolation, cDNA synthesis, and quantification of *DAO* and *DAOA* mRNA levels using qRT-PCR

RNA was isolated from transfected SH-SY5Y, 1321N1, and HEK293 cells using RNeasy Plus Mini Kit (74136, Qiagen) according to manufacturer's guidelines. Firstly, the transfected cells were lysed directly in 6-well plates, and were disrupted and homogenized in the lysis buffer provided in the kit using the TissueLyser II (Qiagen). Secondly, the homogenized lysates were added to the gDNA Eliminator spin column to remove the genomic DNA. Finally, the lysates were added to the RNeasy spin column and RNA was eluted. We used a spectrophotometer (NanoVue Plus, GE Healthcare Life Sciences) to measure RNA concentrations, A260/A280, and A260/A230 ratios. We reverse transcribed 500 ng of RNA using iScript™ cDNA Synthesis Kit (Bio-Rad) as per manufacturer's protocol. In a subset of samples, negative controls were prepared with RNA without reverse transcriptase enzyme. Quantitative real-time reverse transcription-PCR (qRT-PCR) was performed using cDNA, QuantiFast SYBR Green PCR kit (Qiagen), 1 µM DAO or DAOA primers, and reference genes [β-actin (ACTB; QT01680476), ribosomal protein L13a (RPL13A; QT00089915), glyceraldehyde-3-phosphate dehydrogenase (GAPDH; QT01192646), peptidylprolyl isomerase A (PPIA; QT00866137), and ribosomal RNA (R18S; QT00019936); all from Qiagen]. The DAOA primer (QT00058863) was purchased from Qiagen, and the DAO primer was purchased from Microsynth, the sequence of which has been described by (Verrall et al., 2007) (forward primer: CGCAGACGTGATTGTCAACT; reverse primer: GGATGATGTACGGGGAATTG). DAO and DAOA mRNA levels were normalized to the reference genes. LinRegPCR program was used to calculate the PCR efficiencies (Ruijter et al., 2009), and mean PCR efficiencies for all studied amplicons were 91-92%. Normalized *DAO* and *DAOA* mRNA levels were quantified using qBASE plus software (Biogazelle) as described in detail previously (Jagannath et al., 2017).

S2 Protein isolation, Sodium dodecyl sulfate polyacrylamide gel electrophoresis (SDS-PAGE) and Western blot

Protein was isolated from transfected SH-SY5Y, 1321N1, and HEK293 cells using radioimmunoprecipitation assay buffer (RIPA; R0278, Sigma-Aldrich) supplemented with protease inhibitor (04693124001, Sigma-Aldrich) and phosphatase inhibitor (04906845001, Sigma-Aldrich) as per manufacturer's guidelines. The transfected cells were washed twice with ice-cold 1x phosphate buffered saline (PBS) pH 7.4 (10010015, ThermoFisher Scientific). The cells were scraped and collected in RIPA buffer, and were incubated for 30 minutes at 4°C with constant agitation. The cell lysates were centrifuged at 12000 rpm for 10 minutes at 4°C, and supernatants were collected. The protein concentration was determined using the Bradford assay (B6916, Sigma-Aldrich)(Bradford, 1976). Protein lysates (16 µg) were mixed with NuPAGE® LDS Sample Buffer (NP0007, ThermoFisher Scientific) and NuPAGE® Sample Reducing Agent (NP0009, ThermoFisher Scientific), and heated at 70°C for 10 minutes. Then, the lysates were loaded onto NuPAGE™ Novex™ 4-12% Bis-Tris Protein Gels (NP0322BOX, ThermoFisher Scientific), and were subjected to electrophoresis at 180 V for 1 hour in XCell *SureLock*® Mini-Cell (ThermoFisher Scientific). The gels were transferred onto nitrocellulose membranes (IB301002, ThermoFisher Scientific) using iBlot® Gel Transfer Device (ThermoFisher Scientific). The membranes were blocked using 5% non-fat milk (T145.1, Roth) in 1x PBS pH 7.4 with 0.1% Tween-20 (P9416, Sigma-Aldrich) (PBST) for 1 hour at room temperature (RT). After that, the membranes were incubated with primary antibodies against DAO (1:500; ab196563, abcam) or DAOA (1:100; ab205502, abcam) or c-Myc (1:1000; ab32, abcam) or GFP (1:1000; ab1218, abcam) or β-tubulin (1:1000; ab6046, abcam) or vinculin (1:10000; ab129002, abcam) diluted in 5% non-fat milk-PBST at 4°C overnight. The membranes were washed thrice with PBST, and then incubated with anti-rabbit secondary HRP-conjugated antibody (1:2000; ab97051, abcam) or anti-mouse secondary HRP-conjugated antibody (1:2000; ab6789, abcam) for 1 hour at RT. The protein bands were visualized with 20X LumiGLO® Reagent and 20X Peroxide (7003P, Cell Signaling) using ChemiDoc™ XRS+ System with Image Lab™ software (Bio-Rad). The molecular weight of the detected bands was estimated using Novex® Sharp Pre-stained Protein Standard (LC5800, ThermoFisher Scientific). The protein bands were quantified and were normalized to loading controls (β-tubulin or vinculin) using the ImageJ software (Schneider et al., 2012).

1.2 Supplementary Figures

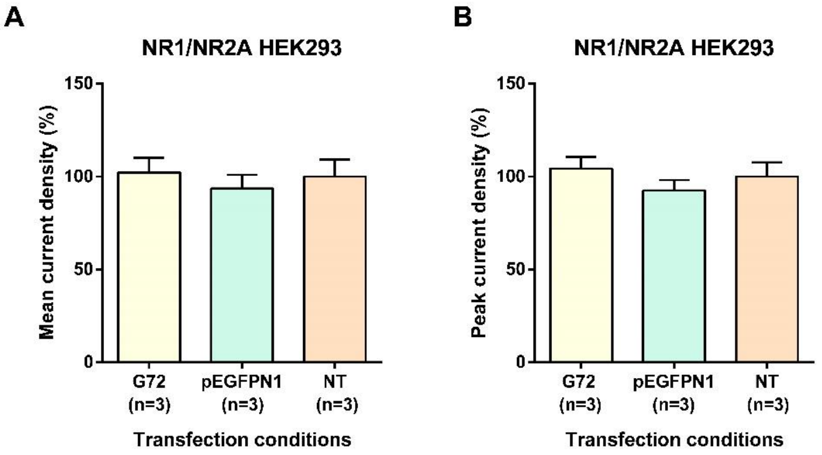

Supplementary Figure S1. NMDA receptor currents in NR1/NR2A HEK293 cells. (**A**) Mean NMDA receptor current across three transfections conditions: G72, pEGFPN1 plasmid, and non-transfected (NT). (**B**) Peak NMDA receptor current across three transfections conditions: G72, pEGFPN1 plasmid, and non-transfected (NT). Data is presented as bar graphs with mean±SEM. Differences between the three transfection conditions was assessed by one-way ANOVA

1.3 Supplementary References

Bradford, M.M., 1976. A rapid and sensitive method for the quantitation of microgram quantities of protein utilizing the principle of protein-dye binding. Anal Biochem 72, 248-254.

Jagannath, V., Marinova, Z., Monoranu, C.M., Walitza, S., Grunblatt, E., 2017. Expression of D-Amino Acid Oxidase (DAO/DAAO) and D-Amino Acid Oxidase Activator (DAOA/G72) during Development and Aging in the Human Post-mortem Brain. Front Neuroanat 11, 31.

Ruijter, J.M., Ramakers, C., Hoogaars, W.M., Karlen, Y., Bakker, O., van den Hoff, M.J., Moorman, A.F., 2009. Amplification efficiency: linking baseline and bias in the analysis of quantitative PCR data. Nucleic Acids Res 37(6), e45.

Schneider, C.A., Rasband, W.S., Eliceiri, K.W., 2012. NIH Image to ImageJ: 25 years of image analysis. Nat Methods 9(7), 671-675.

Verrall, L., Walker, M., Rawlings, N., Benzel, I., Kew, J.N., Harrison, P.J., Burnet, P.W., 2007. d-Amino acid oxidase and serine racemase in human brain: normal distribution and altered expression in schizophrenia. Eur J Neurosci 26(6), 1657-1669.
